# Effect of Ultrasonic-Assisted Casting on Hot Deformation Mechanism and Microstructure of 35CrMo Steel Ingot

**DOI:** 10.3390/ma15010146

**Published:** 2021-12-25

**Authors:** Qiumei Yang, Yajun Zhou, Wei Zhang, Xun Zhang, Mengfei Xu

**Affiliations:** 1 School of Mechanical and Electrical Engineering, Central South University, Changsha 410083, China; Yangqm@csu.edu.cn (Q.Y.); zw340365@csu.edu.cn (W.Z.); jdzhangxun@csu.edu.cn (X.Z.); xumengfei@csu.edu.cn (M.X.); 2 National Key Laboratory of High-Performance Complex Manufacturing, Central South University, Changsha 410083, China; 3 Light Alloy Research Institute, Central South University, Changsha 410083, China

**Keywords:** ultrasonic waves, 35CrMo steel, deformation mechanism, activation energy, work hardening, dynamic recrystallization

## Abstract

Hot compression tests were performed with strain rates (0.01–10 s^−1^) and temperatures (850–1150 °C). The power law relationship between the critical stress and critical strain and Zener–Hollomon parameters was determined by *θ*-*σ* curves. Microstructure was investigated by electron backscattered diffraction. The results showed that the flow behavior and microstructure of 35CrMo steel was affected by ultrasonic-assisted casting. The activation energy of non-ultrasonic and ultrasonic-assisted 35CrMo steel were 410 ± 9.9 and 386 ± 9.4 kJ/mol, respectively, and the activation energy of ultrasonic-assisted specimens was reduced by 6%. In addition, the ultrasonic-assisted treatment refines the grains to some extent and makes the softening process of ultrasonic-assisted samples progress faster, which promoted the development of dynamic recrystallization and the production of *Σ*3 boundaries. The discontinuous dynamic recrystallization was the main DRX nucleation mechanism of the 35CrMo steel.

## 1. Introduction

35CrMo alloy steel is extensively used in the manufacture of important structural components, such as main bearing rotating shafts, vehicle and engine transmission parts and heavy load transmission shafts that work in load environments, because of the excellent impact toughness, high static strength, good hardenability and high fatigue limit [1,2,3,4]. However, many casting defects such as blowholes, inclusions, composition segregation and coarse grains often occur in the process of non-ultrasonic casting, which seriously affect the subsequent forging and properties. In recent years, ultrasonic waves had been widely used in the field of casting because of their cavitation and acoustic flow effect, which refined the solidified structure and avoided pores and inclusions [5,6,7]. Shi et al. [8,9] showed that the introduction of ultrasonic wave in the casting process of 35CrMo steel makes the microstructure change from coarse dendrites to fine dendrites or equi-axed grains and more pronounced grain refinement observed with increasing ultrasonic power. Liu et al. [10] introduced ultrasonic waves into the casting process of AZ91 magnesium alloy and found that fine and uniform non-dendritic structure was obtained under the action of ultrasonic cavitation and flow, and the material exhibited strong yield strength. Shi et al. [11] revealed that the uniform and fine with weakened texture and well-distributed precipitates microstructure obtained by introducing an ultrasonic energy field into the two-roll casting process of 8011 aluminum alloy effectively improves the tensile and yield strength. Kotadiaet al. [12] found that the effective grain refinement and the dendrite transition to equi-axed grains of hypoeutectic Al-Si alloy under the ultrasonic radiation, which was attributed to the enhanced nucleation under ultrasound, causes eutectic splitting to occur under strong fluid flow caused by cavitation. Zhao et al. [13] found that ultrasonic-assisted casting can refine the grains and α-Mg phase of twin-roll casting magnesium alloy and promote the grains to change from dendrite to spherical, improving the tensile strength and elongation. Han et al. [14] investigated the effect of high-intensity ultrasound on the microstructure of Al-Ti-B master alloy in the fabrication and remelting process and found that cavitation and flow field effect of ultrasonic improved the morphology of TiAl_3_ phase and grain refinement, which improved the properties of Al-Ti-B master alloy. The above research showed that the ultrasonic-assisted casting can obtain the fine and uniform solidification microstructure, but the research on the hot deformation and its microstructure is still very little.

Studies showed that the initial microstructure has an important effect on dynamic recrystallization (DRX) behavior during hot deformation, while DRX, dynamic recovery (DRV), grain boundary sliding and grain rotation were related to deformation mechanism and microstructure evolution [15]. The effect of chemical composition of alloys, hot deformation parameters [16,17,18] and initial grain size [19,20,21,22] on the hot deformation behavior and DRX of alloys was reported in recent years. Chen et al. [23] found that the initial grain size had a significant influence on DRX behavior of 3003 aluminum alloy, the critical recrystallization strain decreased with decreased grain size, which was more conducive to the initiation of DRX and promoted the thermoplastic deformation. Wahabi et al. [24] studied the effect of initial grain size on DRX of high purity austenite stainless steels, and larger initial grain sizes promoted a delay in the DDRX onset. Yang et al. [25] investigated the hot deformation and microstructure change of AZ31 magnesium alloy with initial grain sizes of 22 and 90 μm, respectively, during hot compression and found that the initial grain size had obvious influence on the flow curve and grain refinement kinetics. The above studies discussed the effects of initial microstructure on hot deformation and DRX with different processes. However, the influences of microstructures introduced after ultrasonic-assisted casting on high temperature deformation behavior of alloy steels, especially the work hardening properties and microstructure evolution, have not been reported.

The present study focuses the investigation of the hot flow behavior and microstructure evolution of 35CrMo ingot after ultrasonic-assisted casting. The microstructure of 35CrMo steel after ultrasonic-assisted casting was studied by optical microscope (OM) and scanning electron microscope (SEM). The critical parameters of DRX depended on the work hardening curve, and the correlation between the critical stress (*σ*_c_), critical strain (*ε_c_*) and Zener–Hollomon (Z) parameter was established. In addition, EBSD was used to analyze the evolution of deformed microstructure, the characteristics of grain boundary and misorientation angle and the characterization of DRX in detail.

## 2. Experimental Materials and Methods

**Figure 1:**
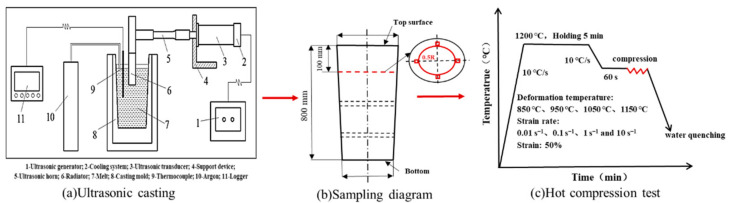
Detailed experimental process of tests: (**a**) ultrasonic casting of 35CrMo steel, (**b**) sampling diagram and (**c**) hot compression test.

The raw materials of 35CrMo steel were heated in resistance furnace (CH Hua, Changsha, China) until melting, gas and slag removal, and kept at 1560 °C for 300 s. Casting was carried out in two ways, A and B. Test A was conducted without ultrasonic-assisted casting. The molten steel was poured directly into the sand mold, the covering agent was added to the molten steel surface after casting and the ingot was cooled in air in the mold. Test B was carried out with ultrasonic-assisted casting. The molten steel was cast into the sand mold, and the ultrasonic wave was introduced from the top of the melt after adding the covering agent for 12 min (the frequency of ultrasonic wave was 20 kHz, the output power was 800 W). Finally, the ingot was cooled in air in the mold. Figure 1a shows a schematic diagram of ultrasonic-assisted casting. Ultimately, two cylindrical 35CrMo alloy ingots with 440 mm diameter and 880 mm height were obtained, with the chemical compositions given in Table 1.

The microstructure specimens of the ingot were circular pieces 15 mm thick, cut from the 100 mm at the top of the ingot (Figure 1b). The macro–microstructure specimens were mechanically corroded in a solution of HNO_3_. Then, the microstructure samples (20 mm× 20 mm) were taken at the edge and 0.5 radius (R) of the ingot (Figure 1b), respectively, and the compressed specimens (Φ 10 mm × 15 mm) were obtained at 0.5 R. For OM and SEM observations, the specimens were etched with a C_2_H_5_OH solution of 4% HNO_3_.

Hot compression tests were carried out on a Gleeble-3810 (San Francisco, America) at the deformation temperatures of 850, 950, 1050 and 1150 °C and strain rates of 0.01, 0.1, 1, 10 s^−1^, and the relative reduction was 50%. The specimens were shallowly grooved at both ends, and a solid lubricant of graphite was added to reduce the friction generated during compression. All specimens were heated at 10 °C/s and held for 5 min before loading and promptly water-quenched after compression (Figure 1c). The hot deformed specimens were sliced along the compression axis along the middle plane and the microstructure of samples was revealed by EBSD. All the samples were mechanically polished and then ion etched by ion etch instrument (Leica RES101, Oberkochen, Germany). The EBSD scans with a step size of 1 μm and a scan area of 450 μm × 450 μm were performed on an Oxford Instruments MAIA3 model EBSD system operated at 30 kV. The scan data were exported to Channnel5 software for post-processing analysis.

## 3. Results and Discussions

### 3.1. Macrostructure and Microstructure Cast by Ultrasonic Assisting

**Figure 2:**
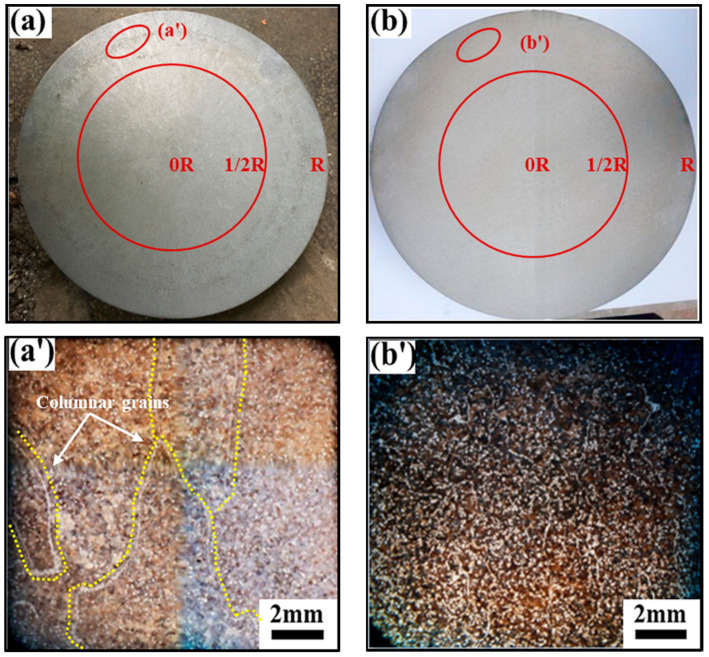
Macro- and microstructure of the ingot: (**a**,**a′**) without ultrasonic; (**b**,**b′**) with ultrasonic.

The microstructure of the ingot is displayed in Figure 2. Figure 2a,a′ reveals that the grain size distribution of the specimens without ultrasonic-assisted casting was coarse, and the obvious columnar grains that develop perpendicular to the center of the ingot shape can be seen. In addition, some thick columnar and equi-axed grains were found in the central region of the ingot, owing to the dendritic branches of columnar grains being broken and drifting to the center to become the nucleus of the remaining metal liquid, which formed coarse equi-axed grains at the center.

**Figure 3:**
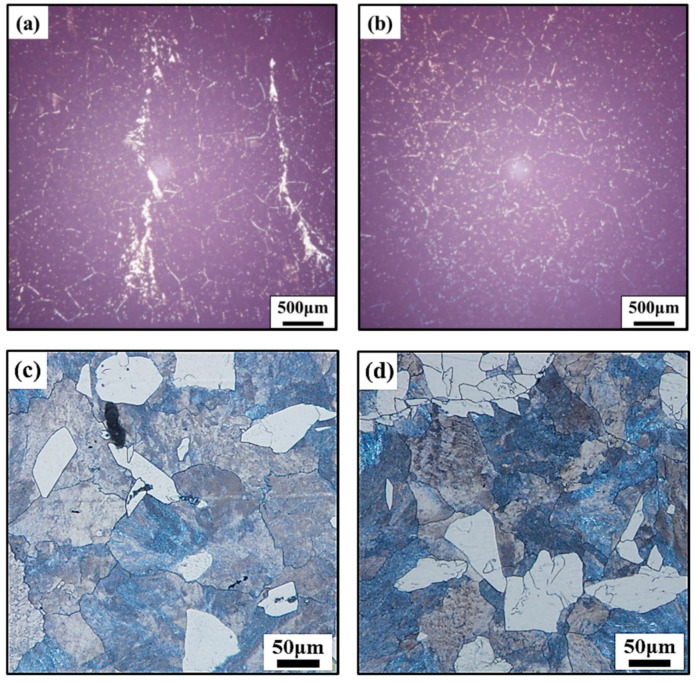
The SEM and OM images of 35CrMo steel ingot at 1/2R in Figure 1: (**a**,**c**) without ultrasonic, (**b**,**d**) with ultrasonic.

However, Figure 2b shows that the grains at the edge of the ultrasonic-assisted casting ingot were equi-axed grains (Figure 2b′), and the coexistence of equi-axed grains and dendrite cell boundaries were observed in the 0.5 R region (Figure 3b). Besides, small equi-axed grains were found in the central region, which indicates that introducing ultrasonic waves can effectively improve the grain uniformity of 35CrMo steel ingot. This was attributed to the stronger shock wave that came into being when the cavitation bubble generated by the high-power ultrasonic cavitation process ruptures, and the crystal embryo reaching the critical dimension is broken by the wave into a plurality of smaller crystal embryos [7]. Therefore, under the effect of ultrasonic cavitation, the fine and uniform solidified structure was finally obtained when the super cooling degree increased, the number of embryos increased and the size of embryos decreased.

The microstructure of Figure 3 was taken from the 0.5 R region of the circular ingot in Figure 2a,b. From Figure 3a, an obvious phenomenon of precipitation phase enrichment of non-ultrasonic specimens was observed, and a bar-like grain boundary appears, while the precipitation phase distribution of that ultrasonic-assisted specimen is uniform (Figure 3b). In addition, Figure 3c,d shows that the casting microstructure was mainly composed of pearlite and ferrite. Furthermore, the grain size was measured and counted several times by the artificial intercept method in OM; the average size of the grains obtained by the non-ultrasonic casting was about 157 ± 1.9 μm, while the ultrasonic-assisted specimens were about 102 ± 1.3 μm, which was about 34.9% lower than the non-ultrasonic assisted. It indicates that introducing ultrasonic waves can refine grains and promote the uniform distribution of precipitated phase of the 35CrMo steel ingot.

### 3.2. Hot Deformation Behavior and Deformation Mechanism

#### 3.2.1. The Stress–Strain Behavior

**Figure 4:**
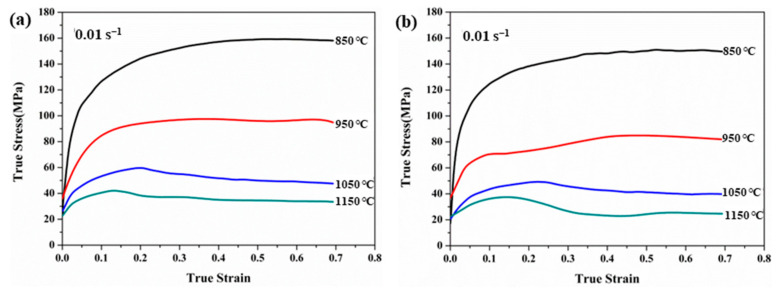
True stress–strain curves of 35CrMo ingots at strain rate of 0.01 s^−1^ and different cast conditions: (**a**) without ultrasonic, (**b**) with ultrasonic.

The true stress–true strain curve of 35CrMo steel cast by non-ultrasonic assisting and ultrasonic assisting under different deformation conditions is shown in Figure 4. At the same deformation condition, the flow stress of the two specimens increased with increased strain, and the strongest flow softening behavior occurred at 850 °C and decreased with increasing deformation temperature. The obvious steady state rheological characteristics were observed in all flow curves when the strain exceeded a certain value. The flow curve is characterized by three stages of work hardening (WH), flow softening (DRV and DRX) and steady state equilibrium [26,27]. In the initial stage of deformation, the flow stress rapidly increased to the critical value, and the number of dislocations increases and accumulates continuously, leading to WH [28,29]. In the softening stage, DRX starts after the deformation cumulative dislocation density outstrips the *ε_c_*, and the nucleation and growth of the new DRX grains lead to dislocation annihilation and decrease the flow stress. The competition between WH and dynamic softening mechanisms results in the evolution of microstructure during hot deformation [30,31,32]. In addition, the heat-softening effect made by DRX and DRV in the softening stage is ultimately stronger than the effect of WH [33]. Finally, the dynamic equilibrium is attained between WH and dynamic softening, and the flow stress reaches steady state [34].

The true stress of the non-ultrasonic-assisted samples was slightly higher than the ultrasonic assisted under the same hot deformation condition as was shown in Figure 4. The reason is that ultrasonic assisted can reduce the grain size of the cast structure, which makes DRX and DRV more likely to occur under high temperature deformation, and the materials with finer grains were softer and easier to be formed. Meanwhile, the flow behavior was obviously affected by the deformation temperature at the constant strain rates. The flow stress decreased with increasing deformation temperature, as was shown in Figure 4. When the deformed temperature rises from 850 to 1050 °C, the true stress difference between ultrasonic assisted and non-ultrasonic in the steady state was larger, which was up to 60 MPa. When the deformed temperature was increased from 1050 to 1150 °C, the true stress difference in the steady state was gradually reduced to about 10 MPa. The reason is that the hot activation of the material was enhanced with the increased deformation temperature, and the average kinetic energy and diffusion rate of metal atoms increased. Furthermore, the nucleation and growth of the DRX grains consume a large number of dislocations [35], resulting in the enhancement of softening [36], and the true stress decreased with increased deformation temperature.

#### 3.2.2. Deformation Activation Energy

The analysis of the data with different processing conditions shows the following relationship between the flow stress, strain rate and deformation temperature of the alloy [28,37,38].

(1) $$\dot{\varepsilon }=A_{1}\sigma^{N}\exp\left(-\frac{Q}{RT}\right)\;\;\;\;\;\alpha \sigma <\;0.8$$

(2) $$\dot{\varepsilon }=A_{2}\exp\left(\beta \sigma \right)\exp\left(-\frac{Q}{RT}\right)\;\;\alpha \sigma >\;1.2$$

(3) $$\dot{\varepsilon }=A{\left[\sinh\left(\alpha \sigma \right)\right]}^{n}\exp\left(-\frac{Q}{RT}\right)\;\;\mathrm{For}\;\mathrm{all}\;\sigma$$

where $\dot{\varepsilon }$ is strain rate; *σ* is flow stress; A_1_, A_2_, A, N, n and *α* are material constants; R is a molar gas constant (R *=* 8.314 J/(mol∙K)); *T* is the deformation temperature; Q is the deformation activation energy, which is a vital parameter of the material with hot deformation. According to Equations (1) and (2), the $\ln\dot{\varepsilon }-\sigma$ and $\ln\dot{\varepsilon }-\ln\sigma$ graphs were drawn according to the experimental data (Figure 4) of thermal deformation and the values of N and *β* were obtained by linear fitting. Then, *α* = *β*/N. The α values of non-ultrasonic casting and ultrasonic-assisted casting 35CrMo steel are *α*_1_ = 0.0071 and *α*_2_ = 0.0072, respectively.

When the strain rate is constant, it is assumed that the deformation activation energy (*Q*) remains constant over a small temperature range, and it can be calculated according to Equation (4) as follows [38]:

(4) $$Q=R{\left.\frac{\partial \ln\dot{\varepsilon }}{\partial \ln\left[\ln\sinh(\alpha \sigma )\right]}\right|}_{T}{\left.\frac{\partial \ln\sinh(\alpha \sigma )}{\partial \ln(1/T)}\right|}_{\dot{\varepsilon }}$$

**Figure 5:**
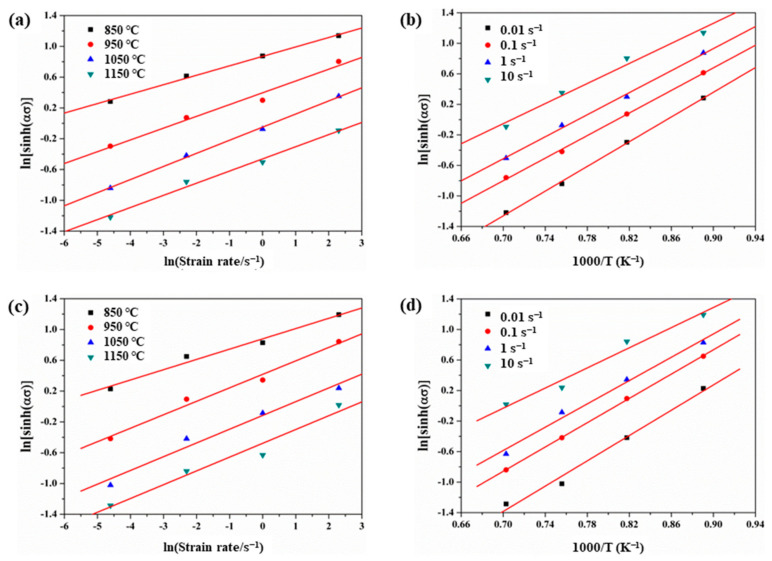
Relationship between peak stress and strain rate of 35CrMo steel during high temperature plastic deformation: (**a**,**b**) without ultrasonic, (**c**,**d**) with ultrasonic.

Therefore, in line with the relationship between $\ln\dot{\varepsilon }-\ln\left[\sin h\left(\alpha \sigma \right)\right]$ and $\ln\left[s\mathrm{inh}\left(\alpha \sigma \right)\right]-1/T$ (Figure 5), the activation energy of non-ultrasonic and ultrasonic-assisted 35CrMo steel was obtained by Equation (4), Q_1_ = 410 ± 9.9 and Q_2_ = 386 ± 9.4 kJ/mol. The results show that ultrasonic-assisted casting can reduce the activation energy ratio of hot working deformation of ingot, reduce WH and promote deformation. This is because the initial grain size of ultrasonic samples is small and grain boundaries were formed more, and the grain boundaries sliding and migration were active at high temperature deformation.

#### 3.2.3. Work Hardening Characteristics

**Figure 6:**
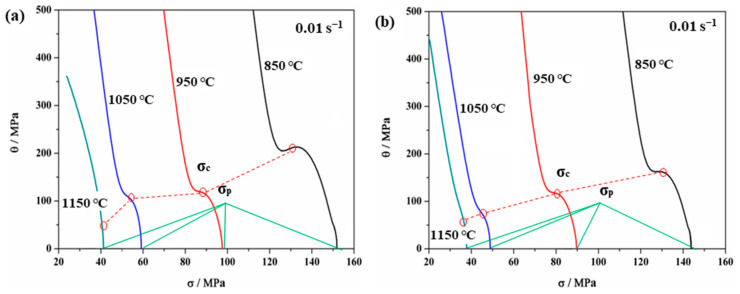
Relationship between *θ* and *σ* of 35CrMo steel under 0.01 s^−1^: (**a**) without ultrasonic, (**b**) with ultrasonic.

**Figure 7:**
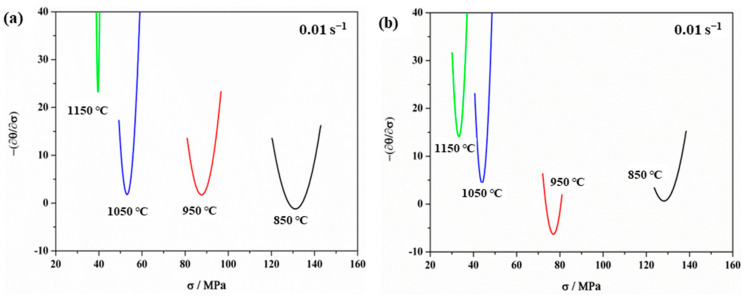
Relationship between −*∂σ*/*∂ε* and *σ* of 35CrMo steel at 0.01 s^−1^ with different deformation temperatures: (**a**) without ultrasonic, (**b**) with ultrasonic.

When the strain rates and deformation temperatures are constant, the rate of change of stress and strain is called the work hardening rate (*θ* = −*∂σ*/*∂ε*), which can be used to identify the WH behavior [33,39]. In addition, the ε*_c_* and *σ_c_* are important parameters for characterizing the DRX initiation and evolution process, which can be determined according to the shape of the WH curve [30]. On the basis of considering irreversible thermodynamics, some scholars consider that the turning point of the *θ*-*σ* curves means the occurrence of DRV and DRX, and the inflection point is *σ*_c_ [40,41]. Figure 6 shows the *θ*-*σ* curves with the inflection points corresponding to the minimum point in the curve of Figure 7. Combined with the analysis of Figure 6 and Figure 7, both the WH curves show a consistent trend independently of the non-ultrasonic or ultrasonic-assisted casting. The WH rates with real stress in two figures were reduced due to the emergence of DRV and DRX at a strain rate of 0.01 s^−1^. However, the *θ* of the ultrasonic-assisted specimens at the inflection point was smaller than the non-ultrasonic specimens at the same processing conditions, which is consistent with the trend of the flow curve. In addition, *σ_c_* was strongly sensitive to deformation temperature, and the *σ_c_* decreased with increased deformation temperature at a strain rate of 0.01 s^−1^ for both the non-ultrasonic specimens and the ultrasonic-assisted specimens. The reason is that the diffusion kinetics were enhanced at a higher deformation temperature, and the hindrance of dislocation motion and grains slip was reduced [37], making DRX more likely to occur.

Studies have shown that the *θ*-*σ* plot exhibits either two [42] or three stages [29]. The behavior of 35CrMo steel in the present study exhibits three stages. The value of *θ* rapidly dropped in the initial stage until a significant inflection point (*σ_c_*) occurred as shown by the WH curves of ultrasonic-assisted cast samples deformed at 850 °C with a strain rate of 0.01 s^−1^, which is an obvious manifestation of the progress of DRX. The increasing and accumulation of the dislocation density during further straining resulted in dynamic enhancement of the substructure which reduced the WH rate. Finally, the *θ* value is zero when it reaches the peak stress (*σ_p_*). Then, the flow stress goes into steady state stress with the strain increasing (Figure 4), which indicates a dynamic balance between the work hardening and dynamic softening mechanisms [30].

The Zener–Hollomon (*Z*) parameter, which the physical meaning is the temperature compensation factor of 35CrMo steel, is obtained from Section 3.2 (*Z*_1_ is for non-ultrasonic casting and *Z*_2_ is for ultrasonic casting):

(5) $$Z_{1}=\dot{\varepsilon }\left(\frac{410\times 10^{3}}{RT}\right)$$

(6) $$Z_{2}=\dot{\varepsilon }\left(\frac{386\times 10^{3}}{RT}\right)$$

**Figure 8:**
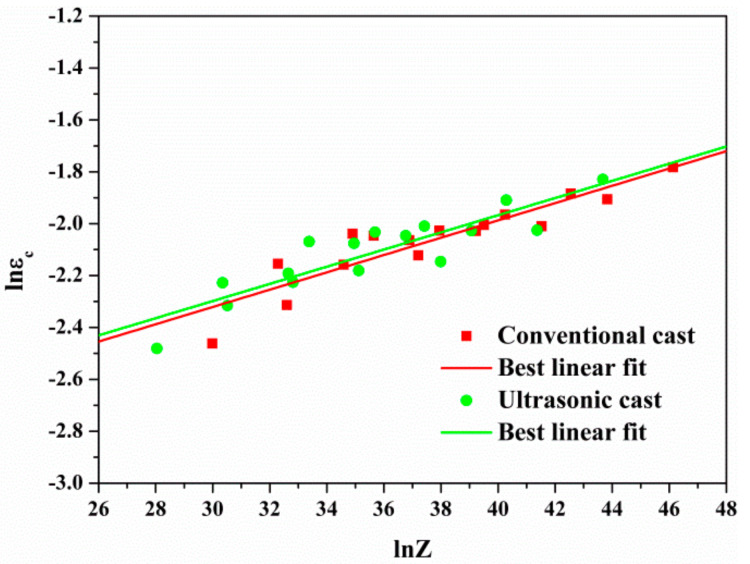
Relationship between ln*ε_c_* and ln*Z* parameter.

The influence of deformation temperature *T* and deformation rate $\dot{\varepsilon }$ on the *ε_c_* of alloy recrystallization was analyzed by introducing *Z* parameters. According to the flow stress curve obtained *ε_c_*, the ln*ε_c_* − ln*Z* plot was plotted (Figure 8), and the following relationships were obtained (ε*_c_*_1_ represents non-ultrasonic casting, ε*_c_*_2_ represents ultrasonic-assisted casting):

(7) $$\ln\varepsilon_{c1}=0.33\ln Z_{1}-3.32$$

(8) $$\ln\varepsilon_{c2}=0.33\ln Z_{2}-3.29$$

Therefore, the critical conditions for DRX of 35CrMo steel in non-ultrasonic casting and ultrasonic-assisted casting were:

(9) $$\varepsilon \geq \varepsilon_{c1}=3.61\times 10^{-2}\ln Z_{1}{}^{0.33}$$

(10) $$\varepsilon \geq \varepsilon_{c2}=3.72\times 10^{-2}\ln Z_{2}{}^{0.33}$$

According to the above analysis, the *ε_c_* of DRX produced by non-ultrasonic casting of 35CrMo steel was more sensitive to the process parameters than the ultrasonic-assisted casting. In addition, the *ε_c_* of DRX of non-ultrasonic specimens was slightly larger than the ultrasonic-assisted casting, which was due to the fine and uniform microstructure of the ultrasonic-assisted casting samples. In addition, the critical recrystallizing strain required was small so that DRX could be sufficiently carried out to promote the thermoplastic deformation of the 35CrMo steel. Simultaneously, the *ε_c_* decreased with increasing deformation temperature and increased with increasing strain rates. This was attributed to the increase in hot oscillation and the diffusion rate of atoms in the alloy with increasing deformation temperature, which promoted the cross-slip and climbing of dislocations, and the more likely DRX occurs, reducing the critical strain of DRX. In addition, the slip and climbing process of dislocations became shorter with increasing strain rate, increasing the number of nucleation and grain growth rate of DRX, which makes the DRX softening obstructed and the critical strain variables for balance of DRX softening and strain hardening increased.

### 3.3. Hot Deformation Microstructure

#### 3.3.1. Microstructure Evolution

**Figure 9:**
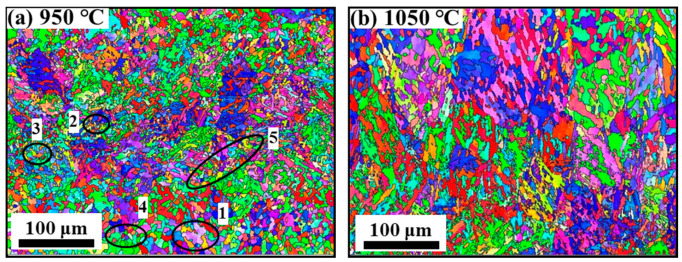

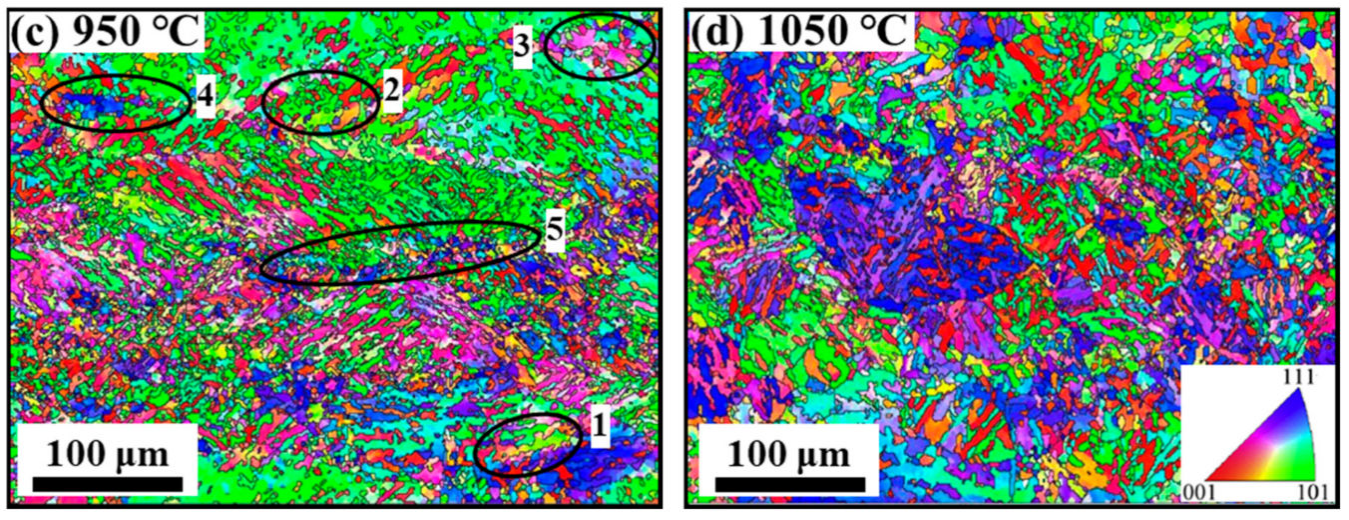
IPF maps of samples deformed at 0.1 s^−1^ at different deformation temperatures of (**a**) without ultrasonic 950 °C, (**b**) without ultrasonic 1050 °C, (**c**) with ultrasonic 950 °C, (**d**) with ultrasonic 1050 °C.

Figure 9 shows the inverse pole figure (IPF) diagram of the non-ultrasonic casting and ultrasonic-assisted casting specimens deformed at 950 and 1050 °C with a strain rate of 0.1 s^−1^. The specimens experienced different degrees of DRX in all deformation conditions studied. Tt was difficult to distinguish between the deformed and DRX grains at a strain rate of 0.1 s^−1^. Whether non-ultrasonic or ultrasonic-assisted specimens, the growth of edge grains was observed. The coarsening degree of grain increased gradually with increasing deformation temperature at the same strain rates, and the deformed grains were elongated. This was due to the increase in diffusion kinetics at high temperature, which made the dislocation movement more intense, and the grain boundary mobility increased, which was beneficial to DRX, so that the small grains were continuously grown over by larger grains to promote grain growth [38,43]. Furthermore, the necklace structure was observed at Figure 9 and suggests that the DRX was incomplete. In addition, at the same deformation conditions, the grains obtained by ultrasonic-assisted samples were smaller.

DDRX with significant nucleation and growth stages is most commonly found in middle and low stacking fault energy materials (SFE) [44]. The main characteristic of DDRX during hot deformation is that the DRX grains are distributed on the grain boundaries of the original coarse grains in the form of a necklace type structure [45,46,47]. However, the serrated grain boundaries, usually with a high local misorientation or strain gradient, are the initial manifestation of DDRX and the potential nucleation center of DRX [48], which can be formed by the grain boundaries bulging as a potential location for subsequent nucleation [43]. In general, grain boundaries bulging is related to strain-induced grain boundaries migration, and the bulging grain boundaries will become the DRX nucleation site [49]. The initiation of DRX is, firstly, the appearance of serrated grain boundaries and then the grain boundaries migrate and transform into convex grain boundaries during the strain induction process [50] and become the core of DRX initiation. Figure 9 shows the different stages of DRX. The serrated grain boundaries are at location 1 of Figure 9a,c, the form of nuclei around the grain boundaries and serrated grain boundaries is observed at positions 2 and 3 and the grown DRX grains are shown at position 4 with the nucleation and growth of DRX. The DRX grains form on the original grain boundaries in a necklace type structure as shown in location 5.

**Figure 10:**
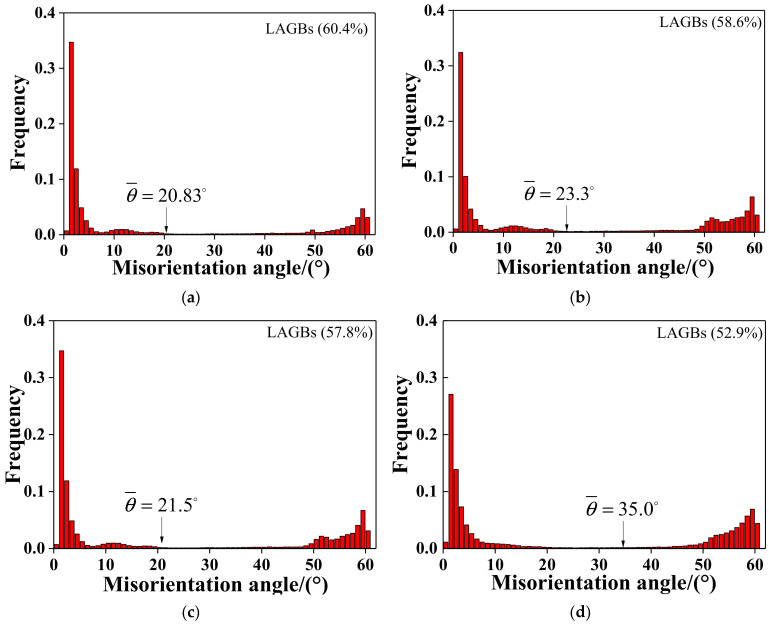
Distribution of grain boundary misorientation angles: (**a**) without ultrasonic 950 °C, 0.1 s^−1^; (**b**) without ultrasonic 1050 °C, 0.1 s^−1^; (**c**) with ultrasonic 950 °C, 0.1 s^−1^; (**d**) with ultrasonic 1050 °C, 0.1 s^−1^.

Figure 10 and Table 2 show the misorientation distribution at different deformation temperatures at a strain rate of 0.1 s^−1^. Some studies suggested that the DRX process can be understood as establishing a dynamic balance from low angle grain boundaries (LAGBs, 2 ≤ *θ* ≤ 10°) to high angle grain boundaries (HAGBs, *θ* > 15°), the strain energy storage produced during hot deformation prompts the growth and migration of HAGBs, which promotes dislocation motion, and LAGBs are continuously consumed with the dislocation annihilation [51,52]. From Figure 10a,c, the fraction of LAGBs was high at 950 °C for both the non-ultrasonic or ultrasonic samples at the same processing condition, and the values are 60.4 and 57.8%, respectively. Furthermore, most of the LAGBs occur near the mother grain boundaries and the twin boundary (Figure 9a,c), which is attributed to the formation of dislocation proliferation and dislocation boundary [43]. Meanwhile, the fraction of LAGBs gradually decreased with increased temperature while HAGBs increased gradually, and the average misorientation angle ($\bar{\theta }$) increased with increased temperature owing to the sufficient energy and time at high deformed temperature for the LAGBs to disappear or merge into HAGBs [53,54,55]. It indicates that the LAGBs transition to HAGBs and the dynamics of DRX is enhanced with increasing deformation temperature. In addition, it is well known that an increase in the fraction of misorientation 10°–15° medium angle grain boundaries (MAGBs) means the occurrence of continuous dynamic crystallization (CDRX) [43]. As shown in Figure 10 and Table 2, MAGBs had no significant change with increased deformation temperature for both non-ultrasonic or ultrasonic-assisted samples, and the distribution of the misorientation difference exhibits bimodal characteristics. The results show that LAGBs gradually change to HAGBs grain boundary in a discontinuity way, indicating that the main DRX mechanism was DDRX [56]. The $\bar{\theta }$ value of ultrasound-assisted specimens is significantly larger than the non-ultrasonic ones when the deformation temperature is raised from 950 to 1050 °C, indicating that the smaller grain microstructure (Figure 3) of the ultrasonic-assisted samples is more conducive to the conversion of LAGBs to HAGBs, forming more nucleation sites, which makes it more prone to DRX and refined the microstructure [24].

#### 3.3.2. Coincidence Site Lattice Characteristics

**Figure 11:**
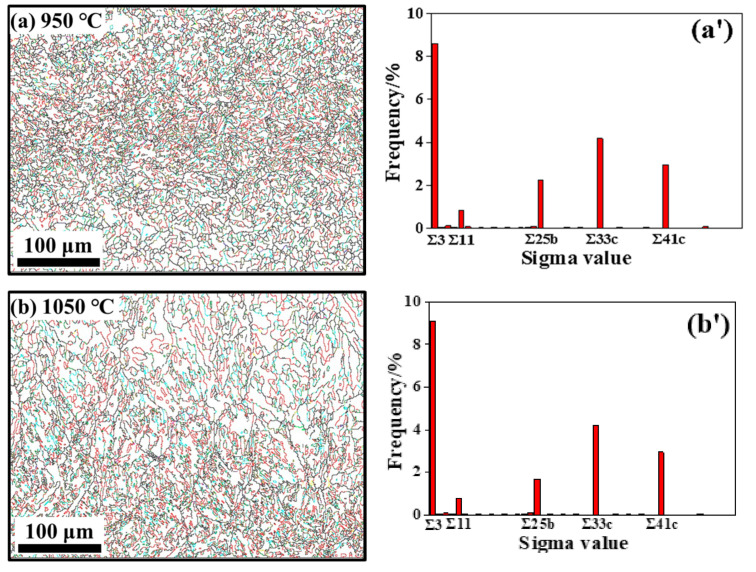

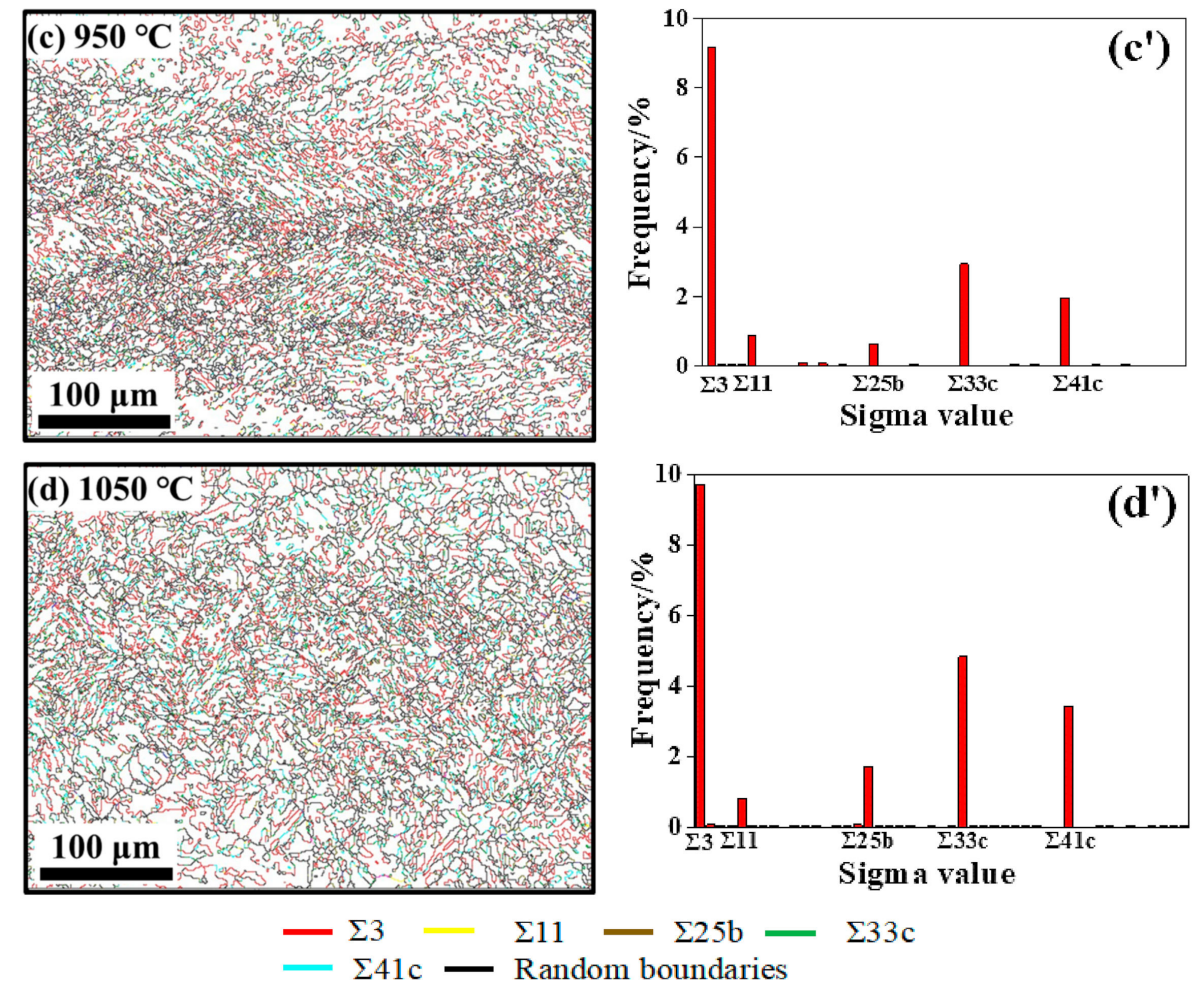
CSL boundary distribution at a strain rate of 0.1 s^−1^ and different deformation temperatures of 950, 1050 °C: (**a**,**a′**,**b**,**b′**) without ultrasonic and (**c**,**c’**,**d**,**d’**) with ultrasonic.

A coincidence site lattice (CSL) grain boundary is a kind of high-angle boundary with special axial direction and directional misalignment angles, where the microstructure of the material is obviously affected [57,58,59]. The detailed information on the different CSL boundary fractions of non-ultrasonic and ultrasonic-assisted specimens deformed at 950 and 1050 °C with strain rates of 0.1 s^−1^ are displayed in Figure 11. The main CSL boundary types after hot deformation of non-ultrasonic and ultrasonic samples were *Σ*3, *Σ*11, *Σ*25b, *Σ*33c, *Σ*41c, and the fraction of *Σ*3 boundaries is the largest, obviously, which is associated with the volume fraction trend of HAGBs and DRX. This may be attributed to a grain growth in the DRX process or grain boundary migration that promotes the interaction between the original *Σ*3 interface, resulting in a new *Σ*3 interface [51]. In addition, the existence of new nuclei in the DRX process also promotes the formation of *Σ*3 boundary, and the newer DRX boundary is produced, the more *Σ*3 boundary there is [59]. On the other hand, the *Σ*3 boundary fraction of the non-ultrasonic samples increased, and the fractions of *Σ*11, *Σ*25b, *Σ*33c, *Σ*41c remained basically at the same level, while the fractions of ultrasonic specimens *Σ*3, *Σ*25b, *Σ*33c, *Σ*41c all showed an evident increasing trend can be observed for deformed from 950 to 1050 °C and a strain rate of 0.1 s^−1^, indicating that the grain boundaries migration and DRX ability of ultrasonic specimens are strong at higher deformation temperature, which coincides with Figure 9. This is owing to the smaller initial grain sizes of the ultrasonic specimens lead to more nucleation sites and the softening process progresses faster, which is beneficial to the nucleation and growth of DRX [23,24], resulting in promoting the production of more *Σ*CSL boundaries.

#### 3.3.3. Characterization of Dynamic Recrystallization

**Figure 12:**
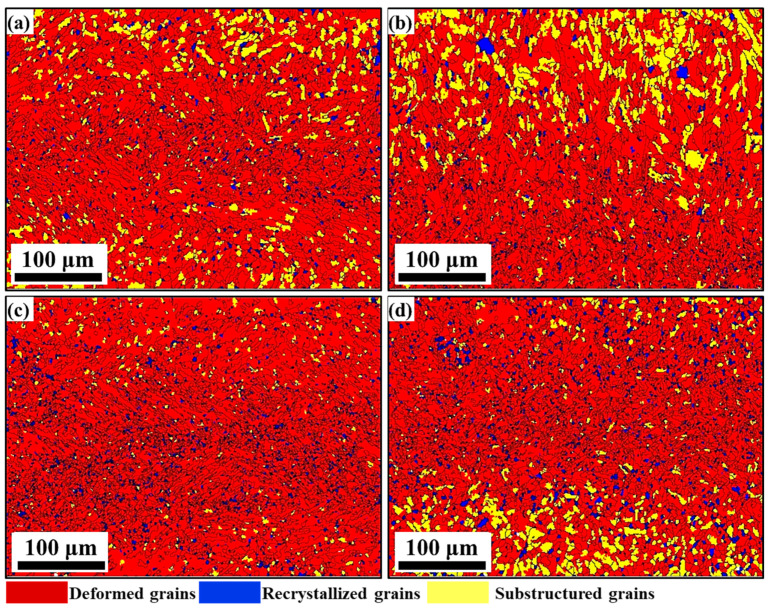
Color IPF maps illustrating the DRX grains (in blue), deformed grains (in red) and sub-grains (in yellow) at a strain rate of 0.1s^−1^ with (**a**) without ultrasonic 950 °C, (**b**) without ultrasonic 1050 °C, (**c**) with ultrasonic 950 °C, (**d**) with ultrasonic 1050 °C.

**Figure 13:**
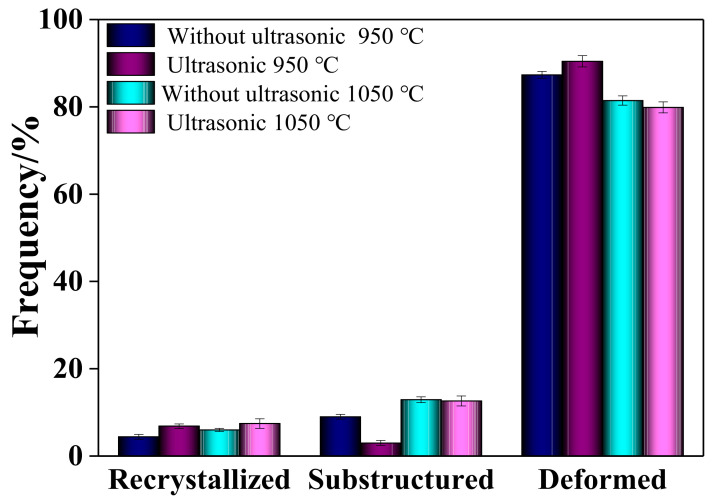
Evolution of deformed grains, sub-grains and DRX grains at a strain rate of 0.1 s^−1^ and different deformation temperatures.

The IPF diagrams of non-ultrasonic and ultrasonic specimens at different deformation temperatures of 0.1 s^−1^ strain rate are given in Figure 12. The deformed, DRX and sub-grains are denoted by red, blue and yellow colors, respectively. The fraction of the deformed grains, sub-grains and DRX grains in Figure 12 was counted by Channnel5 as shown in Figure 13. Combined with the analysis of Figure 12 and Figure 13, the DRX was enhanced with increased deformation temperatures, and the DRX grains and sub-grains gradually increase whether without or with ultrasonic-assisted samples. This indicates that DDRX promotes the transformation from deformed grains into DRX grains, increasing the proportion of HAGBs at the expense of LAGBs (Figure 10). Meanwhile, the lower portion of the DRX grains (deformed at 1050 °C) indicates that heat and strain energy were insufficient to promote DRX (Figure 10a,c). In addition, comparing Figure 12a,c and Figure 12b,d, the DRX grains of the ultrasonic samples were increased and finer grains were observed compared with the non-ultrasonic ones, indicating that the DRX of ultrasonic-assisted casting specimens is stronger. These was due to the ultrasonic-assisted specimens with finer initial microstructure and more grain boundaries before deformation, which is beneficial to provide more nucleation sites. Therefore, the DRX rate of ultrasonic samples is faster than the non-ultrasonic, and a large number of nuclei in the fine-grained ultrasonic samples result in a finer and uniform microstructure.

**Figure 14:**
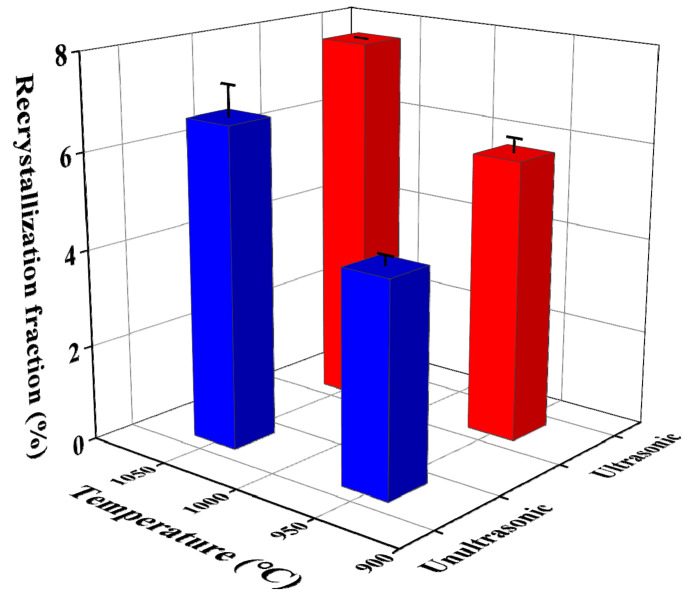
The evolution of DRX volume fraction at a strain rate of 0.1 s^−1^ and different deformation temperatures.

The DRX fraction of non-ultrasonic and ultrasonic specimens at different temperatures at a strain rate of 0.1 s^−1^ is shown in Figure 14. The DRX fractions of without and with ultrasonic samples deformed at 950 °C are 4.4 and 5.8%, respectively, indicating the DRX was lower and coincided with the deformation microstructure in Figure 12, which is attributed to the shorter deformation time at lower temperatures. After deformation at 1050 °C, the DRX volume fraction of without and with ultrasonic samples increased, up to 6.7 and 7.8%, respectively, indicating that the volume fraction of DRX increased with increasing temperature, which is consistent with the influence of temperature on the flow curve. This was due to the higher heat energy and the larger local storage energy obtained at high temperature, which is beneficial to the migration of grain boundaries and dislocation movement, improves the mobility of grain boundaries and makes it easier for DRX [60,61]. On the other hand, the increase in the proportion of typical CSL interface (especially *Σ*3) plays a certain role in promoting the obvious expansion of DRX degree with the increase in deformation temperatures with a strain rate of 0.1 s^−1^, and the enhanced DRX ability was consistent with the evolution of the low CSL interface. Furthermore, the DRX volume fraction of the non-ultrasonic specimens was smaller than the ultrasonic specimens both deformed at 950 and 1050 °C, which corresponded with the deformation structure observed in Figure 10 and Figure 12.

## 4. Conclusions

The hot deformation behavior and microstructure evolution of the ultrasonic-assisted 35CrMo steel were investigated. The results show that the ultrasonic-assisted casting treatment can effectively control the morphology and size of as-cast grains to a certain extent and help to develop DRX during the subsequent forge. The important conclusions are summarized as follows:

(1) The acoustic streaming and cavitation effects of ultrasonic waves affected the cast solidification microstructure. After ultrasonic-assisted casting, the microstructure changed from columnar grains to equi-axed grains, the distribution of the precipitate was uniform and the grains were refined to a certain extent.

(2) At the same deformation conditions, the initial grain size of ultrasonic-assisted samples was smaller, and the softening process was faster. Besides, the activation energy of non-ultrasonic and ultrasonic-assisted 35CrMo steel was 410 ± 9.9 and 386 ± 9.4 kJ/mol, respectively, and the power law functions between critical parameters (*σ_c_* and ε*_c_*) and Zener–Hollomon parameters were established.

(3) The dynamic recrystallization behavior was influenced by ultrasonic-assisted casting. At the same deformation conditions, the HAGBs, CSL boundary and dynamic recrystallization volume fraction of the ultrasonic-assisted samples were larger, which indicated that the DRX of ultrasonic-assisted samples is stronger. Besides, discontinuous DRX characterized by necklace structure is the main recrystallization mechanism of 35CrMo steel.

## Figures and Tables

**Table 1:** Composition of GB 35CrMo steel (wt %).

Chemical Composition	C	Si	Mn	Mo	S	P	Cr	Fe
Measured	0.34	0.21	0.56	0.19	0.005	0.019	0.95	Bal

**Table 2:** Statistics of grain boundary misorientation angles.

Case	without Ultrasonic		with Ultrasonic	
	950 °C, 0.1 s^−1^	1050 °C, 0.1 s^−1^	950 °C, 0.1 s^−1^	1050 °C, 0.1 s^−1^
$\bar{\theta }$	20.83°	23.3°	21.5°	35.0°
2 ≤ *θ* ≤ 10°	0.604	0.586	0.578	0.529
10°–15°	0.039	0.026	0.04	0.05
>15°	0.357	0.387	0.380	0.421

## Data Availability

The raw/processed data required to reproduce these findings cannot be shared at this time as the data also forms part of an ongoing study.

## References

[1] Yang Q.M., Zhou Y.J., Li Z., Mao D.H. (2019). Effect of Hot Deformation Process Parameters on Microstructure and Corrosion Behavior of 35Crmov Steel. Materials.

[2] Chen J.L., Zhou Y.J., Shi C., Mao D.H. (2017). Microscopic Analysis and Electrochemical Behavior of Fe-Based Coating Produced by Laser Cladding. Metals.

[3] Li Z., Zhou Y.J., Wang S.X. (2018). Influence of Strain and Stress Triaxiality on the Fracture Behavior of Gb 35Crmo Steel During Hot Tensile Testing. Adv. Mater. Sci. Eng..

[4] Li H.B., Chen M.S., Tian Y.Q., Chen L.S., Chen L.Q. (2020). Ultra-Fine-Grained Ferrite Prepared from Dynamic Reversal Austenite During Warm Deformation. Acta Metall. Sin. (Engl. Lett.).

[5] Kumar A., Kumaresan T., Pandit A.B., Joshi J.B. (2006). Characterization of Flow Phenomena Induced by Ultrasonic Horn. Chem. Eng. Sci..

[6] Madelin G., Grucker D., Franconi J., Thiaudiere E. (2006). Magnetic Resonance Imaging of Acoustic Streaming: Absorption Coefficient and Acoustic Field Shape Estimation. Ultrasonics.

[7] Zhang X.P., Kang J.W., Wang S., Ma J.Y., Huang T.Y. (2015). The Effect of Ultrasonic Processing on Solidification Microstructure and Heat Transfer in Stainless Steel Melt. Ultrason. Sonochemistry.

[8] Liang G., Shi C., Zhou Y.J., Mao D.H. (2016). Numerical Simulation and Experimental Study of an Ultrasonic Waveguide for Ultrasonic Casting of 35CrMo steel. J. Iron Steel Res. Int..

[9] Shi C., Li F., Liang G., Mao D.H. (2018). Effect of Ultrasonic Melt Treatment on Microstructure and Mechanical Properties of 35Crmo Steel Casting. IOP Conference Series: Earth and Environmental Science.

[10] Liu X.B., Osawa Y., Takamori S., Mukai T. (2008). Microstructure and Mechanical Properties of Az91 Alloy Produced with Ultrasonic Vibration. Mater. Sci. Eng. A.

[11] Shi C., Shen K. (2018). Twin-Roll Casting 8011 Aluminium Alloy Strips under Ultrasonic Energy Field. Int. J. Light. Mater. Manu..

[12] Kotadia H.R., Das A. (2015). Modification of Solidification Microstructure in Hypo- And Hyper-Eutectic Al–Si Alloys under High-Intensity Ultrasonic Irradiation. J. Alloys Compd..

[13] Zhao J., Yu K., Xue X.Y., Mao D.H., Li J.P. (2011). Effects of Ultrasonic Treatment On the Tensile Properties and Microstructure of Twin Roll Casting Mg–3%Al–1%Zn–0.8%Ce–0.3%Mn (Wt%) Alloy Strips. J. Alloys Compd..

[14] Han Y.F., Li K., Wang J., Shu D., Sun B.B. (2005). Influence of High-Intensity Ultrasound On Grain Refining Performance of Al–5Ti–1B Master Alloy On Aluminium. Mater. Sci. Eng. A.

[15] Liu Z.G., Li P.J., Xiong L.T., Liu T.Y., He L.J. (2017). High-Temperature Tensile Deformation Behavior and Microstructure Evolution of Ti55 Titanium Alloy. Mater. Sci. Eng. A.

[16] He H.L., Yi Y.P., Cui J.D., Huang S.Q. (2019). Hot Deformation Characteristics and Processing Parameter Optimization of 2219 Al Alloy Using Constitutive Equation and Processing Map. Vacuum.

[17] Chen X.M., Lin Y.C., Wen D.X., Zhang J.L., He M. (2014). Dynamic Recrystallization Behavior of a Typical Nickel-Based Superalloy During Hot Deformation. Mater. Des..

[18] Li H.B., Fan L.F., Chen L.S., Jia L.Y. (2019). Effect of cooling mode on the microstructure and mechanical properties of medium carbon steel after warm rolling. Iron. Steel..

[19] Rezaei Ashtiani H.R., Parsa M.H., Bisadi H. (2012). Effects of Initial Grain Size on Hot Deformation Behavior of Commercial Pure Aluminum. Mater. Des..

[20] Tan Y.B., Yang L.H., Duan J.L., Liu W.C., Zhang J.W., Liu R.P. (2014). Effect of Initial Grain Size On the Hot Deformation Behavior of 47Zr–45Ti–5Al–3V Alloy. J. Nucl. Mater..

[21] Wang T., Guo H.Z., Wang Y.W., Peng X.N., Zhao Y., Yao Z.K. (2011). The Effect of Microstructure on Tensile Properties, Deformation Mechanisms and Fracture Models of Tg6 High Temperature Titanium Alloy. Mater. Sci. Eng. A.

[22] Lin Y.C., Jiang X.Y., Shuai C.J., Zhao C.Y., He D.G., Chen M.S., Chen C. (2018). Effects of Initial Microstructures On Hot Tensile Deformation Behaviors and Fracture Characteristics of Ti-6Al-4V Alloy. Mater. Sci. Eng. A.

[23] Chen G.Q., Fu G.S., Wei T.Y., Cheng C.Z., Wang H.S., Wang J.D. (2018). Effect of Initial Grain Size On the Dynamic Recrystallization of Hot Deformation for 3003 Aluminum Alloy. Met. Mater. Int..

[24] El Wahabi M., Gavard L., Montheillet F., Cabrera J.M., Prado J.M. (2005). Effect of Initial Grain Size On Dynamic Recrystallization in High Purity Austenitic Stainless Steels. Acta Mater..

[25] Yang X.Y., Sanada M., Miura H., Sakai T. (2005). Effect of Initial Grain Size on Deformation Behavior and Dynamic Recrystallization of Magnesium Alloy Az31. Mater. Sci. Forum..

[26] Hassani F.Z., Ketabchi M., Ebrahimi G.R., Bruschi S. (2016). Hot Compression Deformation Characteristics and Microstructural Evolution of a Co–Cr–Mo–C Alloy: Effect of Precipitate and Martensitic Transformation. Mater. Sci. Eng. A.

[27] Lin Y.C., Pang G.D., Jiang Y.Q., Liu X.G., Zhang X.Y., Chen C., Zhou K.G. (2019). Hot Compressive Deformation Behavior and Microstructure Evolution of a Ti-55511 Alloy with Basket-Weave Microstructures. Vacuum.

[28] Wen D., Lin Y.C., Li X., Singh S.K. (2018). Hot Deformation Characteristics and Dislocation Substructure Evolution of a Nickel-Base Alloy Considering Effects of δ Phase. J. Alloys Compd..

[29] Wen D., Lin Y.C., Chen J., Chen X., Zhang J., Liang Y., Li L. (2015). Work-Hardening Behaviors of Typical Solution-Treated and Aged Ni-Based Superalloys During Hot Deformation. J. Alloys Compd..

[30] Kumar S.S.S., Raghu T., Bhattacharjee P.P., Rao G.A., Borah U. (2017). Work Hardening Characteristics and Microstructural Evolution during Hot Deformation of a Nickel Superalloy at Moderate Strain Rates. J. Alloys Compd..

[31] Qin X., Huang D., Yan X., Zhang X., Qi M., Yue S. (2019). Hot Deformation Behaviors and Optimization of Processing Parameters for Alloy 602 Ca. J. Alloys Compd..

[32] Li H., Zheng X., Wan D., Chen L. (2019). Effect of time interval on microstructure evolution of medium carbon steel during warm deformation. J. Iron Steel Res. Int..

[33] Kingkam W., Zhao C., Li H., Zhang H., Li Z. (2019). Hot Deformation and Corrosion Resistance of High-Strength Low-Alloy Steel. Acta Metall. Sin. (Engl. Lett.).

[34] Sakai T., Belyakov A., Kaibyshev R., Miura H., Jonas J.J. (2014). Dynamic and Post-Dynamic Recrystallization under Hot, Cold and Severe Plastic Deformation Conditions. Prog. Mater. Sci..

[35] He D.G., Lin Y.C., Wang L.H., Wu Q., Zu Z.H., Cheng H. (2019). Influences of Pre-Precipitated δ Phase On Microstructures and Hot Compressive Deformation Features of a Nickel-Based Superalloy. Vacuum.

[36] Marchattiwar A., Sarkar A., Chakravartty J.K., Kashyap B.P. (2013). Dynamic Recrystallization during Hot Deformation of 304 Austenitic Stainless Steel. J. Mater. Eng. Perform..

[37] Zhang P., Hu C., Zhu Q., Ding C.G., Qin H.Y. (2015). Hot Compression Deformation and Constitutive Modeling of Gh4698 Alloy. Mater. Des..

[38] Xu L., Chen L., Chen G.J., Wang M.Q. (2018). Hot Deformation Behavior and Microstructure Analysis of 25Cr3Mo3Ninb Steel during Hot Compression Tests. Vacuum.

[39] Wu H.Y., Du L.X., Liu X.H. (2011). Dynamic Recrystallization and Precipitation Behavior of Mn-Cu-V Weathering Steel. J. Mater. Sci. Technol..

[40] Poliak E.I., Jonas J.J. (1996). A One-Parameter Approach to Determining the Critical Conditions for the Initiation of Dynamic Recrystallization. Acta Mater..

[41] Quan G.Z., Li G.S., Chen T., Wang Y.X., Zhang Y.W., Zhou J. (2011). Dynamic Recrystallization Kinetics of 42Crmo Steel During Compression at Different Temperatures and Strain Rates. Mater. Sci. Eng. A.

[42] Dehghan-Manshadi A., Barnett M., Hodgson P. (2008). Hot Deformation and Recrystallization of Austenitic Stainless Steel: Part I. Dynamic Recrystallization. Mater. Sci. Trans. A.

[43] Lin Y.C., Wu X.Y., Chen X.M., Chen J., Wen D.X., Zhang J.L., Li L.T. (2015). EBSD Study of a Hot Deformed Nickel-Based Superalloy. J. Alloys Compd..

[44] Rout M., Ranjan R., Pal S.K., Singh S.B. (2018). EBSD Study of Microstructure Evolution during Axisymmetric Hot Compression of 304Ln Stainless Steel. Mater. Sci. Eng. A.

[45] Ghazani M.S., Eghbali B. (2018). Characterization of the Hot Deformation Microstructure of AISI 321 Austenitic Stainless Steel. Mater. Sci. Eng. A.

[46] Wang G.Q., Chen M.S., Li H.B., Lin Y.C., Zeng W.D., Ma Y.Y. (2021). Methods and Mechanisms for Uniformly Refining Deformed Mixed and Coarse Grains Inside a Solution-Treated Ni-based Superalloy by Two-Stage Heat Treatment. J. Mater. Sci. Technol..

[47] Wan Z.P., Hu L.X., Sun Y., Wang T., Li Z. (2018). Microstructure Evolution and Dynamic Softening Mechanisms during High-Temperature Deformation of a Precipitate Hardening Ni-Based Superalloy. Vacuum.

[48] Chen Z.J., Lin Y.C., He D.G., Lou Y.M., Chen M.S. (2021). A unified dislocation density-based model for an aged polycrystalline Ni-based superalloy considering the coupled effects of complicate deformation mechanisms and initial δ phase. Mater. Sci. Eng. A.

[49] Shimizu I. (2008). Theories and Applicability of Grain Size Piezometers: The Role of Dynamic Recrystallization Mechanisms. J. Struct. Geol..

[50] Su G., Yun Z., Lin Y.C., He D.G., Zhang S., Chen Z.J. (2021). Microstructure Evolution and a Unified Constitutive Model of Ti-55511 Alloy Compressed at Stepped Strain Rates. Materials.

[51] He S., Li C.S., Zheng J.J., Ren J.Y., Han Y.H. (2018). Effect of Deformation Temperature On Dynamic Recrystallization and CSL Grain Boundary Distribution of Fe-36%Ni Invar Alloy. J. Mater. Eng. Perform..

[52] Wang M.H., Wang W.H., Zhou J., Dong X.G., Jia Y.J. (2012). Strain Effects On Microstructure Behavior of 7050-H112 Aluminum Alloy During Hot Compression. J. Mater. Sci..

[53] Liu Y.H., Ning Y.Q., Nan Y., Liang H.Q., Li Y.Z., Zhao Z.L. (2015). Characterization of Hot Deformation Behavior and Processing Map of FGH4096–GH4133B Dual Alloys. J. Alloys Compd..

[54] Xia X.S., Chen Q., Li J.P., Shu D.Y., Hu C.K., Huang S.H., Zhao Z.D. (2014). Characterization of Hot Deformation Behavior of as-Extruded Mg–Gd–Y–Zn–Zr Alloy. J. Alloys Compd..

[55] Wen D.X., Lin Y.C., Zhou Y. (2017). A New Dynamic Recrystallization Kinetics Model for a Nb Containing Ni-Fe-Cr-base Superalloy Considering Influences of Initial δ Phase. Vacuum.

[56] Cao Y., Di H.S., Zhang J.Q., Zhang J.C., Ma T.J., Misra R.D.K. (2013). An Electron Backscattered Diffraction Study on the Dynamic Recrystallization Behavior of a Nickel–Chromium Alloy (800H) during Hot Deformation. Mater. Sci. Eng. A.

[57] Brandon D.G. (1966). The Structure of High-Angle Grain Boundaries. Acta Metall..

[58] Odnobokova M., Tikhonova M., Belyakov A., Kaibyshev R. (2017). Development of *Σ*3^n^ CSL Boundaries in Austenitic Stainless Steels Subjected to Large Strain Deformation and Annealing. J. Mater. Sci..

[59] Zhang H.B., Zhang K.F., Zhou H.P., Lu Z., Zhao C.H., Yang X.L. (2015). Effect of Strain Rate On Microstructure Evolution of a Nickel-Based Superalloy During Hot Deformation. Mater. Des..

[60] Lin Y.C., He D.G., Chen M.S., Chen X.M., Zhao C.Y., Ma X., Long Z.L. (2016). EBSD Analysis of Evolution of Dynamic Recrystallization Grains and δ Phase in a Nickel-Based Superalloy During Hot Compressive Deformation. Mater. Des..

[61] Lin Y.C., Huang J., He D.G., Zhang X.Y., Wu Q., Wang L.H., Chen C., Zhou K.C. (2019). Phase Transformation and Dynamic Recrystallization Behaviors in a Ti55511 Titanium Alloy during Hot Compression. J. Alloys Compd..

